# Impact of biologicals on revision ESS numbers in CRSwNP in a tertiary care rhinology center

**DOI:** 10.3389/falgy.2025.1725159

**Published:** 2026-01-29

**Authors:** Judith E. Adriaenssens, An-Sofie Viskens, Elien Borgers, Peter W. Hellings

**Affiliations:** 1 Faculty of Medicine, KULeuven, Leuven, Belgium; 2 Department of Otorhinolaryngology & Head and Neck Surgery, UZA, Antwerp, Belgium; 3 Laboratory of Allergy and Clinical Immunology, KU Leuven, Leuven, Belgium; 4 Department of Otorhinolaryngology & Head and Neck Surgery, UZ Leuven, Leuven, Belgium; 5 Upper Airways Research Laboratory, University of Ghent, Ghent, Belgium

**Keywords:** biologicals, chronic rhinosinusitis with nasal polyps, ESS, revision ESS, tertiary care center

## Abstract

**Background:**

Since 2022, several biologics are indicated and reimbursed for Belgian patients with severe uncontrolled CRSwNP despite previous endoscopic sinus surgery (ESS). Data on the impact of biologics on the number of patients undergoing revision ESS are lacking.

**Methods:**

We analyzed the trend in numbers and percentages of patients with severe uncontrolled CRSwNP being offered primary ESS, revision ESS or biologics according to both academic- and reimbursement criteria at a tertiary care Rhinology center (University Hospitals Leuven) in Belgium, from 2019 until 2023.

**Results:**

A total of 641 patients with severe uncontrolled CRSwNP had been offered primary/revision ESS or biologics in the past 5 years. In contrast to the overall increase in annual numbers of CRSwNP patients treated for uncontrolled CRSwNP from 2019 (*n* = 128) until 2023 (*n* = 160) by ESS or biologics, the absolute number of patients undergoing revision surgery is reduced by the advent of biologics since 2022. Patient numbers undergoing revision ESS were 66 and 59 in 2019 and 2020 respectively, and 48 in 2023. Moreover, the percentage of patients undergoing revision surgery dropped from 52% and 56% in 2019 and 2020, respectively to 34% and 30% in 2022 and 2023, respectively. The percentage of primary ESS procedures remained stable.

**Conclusion:**

The advent of biologics reduced the number and percentage of severe uncontrolled CRSwNP patients undergoing revision ESS, in contrast to number and percentage of primary ESS.

## Introduction

Chronic rhinosinusitis with nasal polyps (CRSwNP) is an inflammatory disease of the nasal and paranasal sinuses, with a prevalence of 3%–6% in adults. It is often associated with other comorbidities, such as asthma and aspirin-exacerbated respiratory disease (AERD), leading to a significant reduced quality of life (QoL) (1, 2).

CRSwNP is diagnosed in patients with nasal polyps who have symptoms of nasal blockage/congestion and/or anterior or postnasal drip with or without impaired sense of smell and facial pain/pressure, for more than 12 weeks (1).

The pathophysiology of CRSwNP is complex and involves a combination of genetic, environmental and immunological factors. In Western populations, CRSwNP is predominantly driven by type 2 inflammation, which is associated with increased resistance to therapy compared to type 1 or type 3 inflammatory profiles (1, 3).

Key molecules in the type 2 immune cascade include type 2 helper cells, interleukin (IL)-4, IL-5 and IL-13. All of which play important roles in increasing local eosinophils and total IgE levels (4).

Nasal rinses and intranasal corticosteroids are the first-line treatment for this disease. When this treatment fails, the next step is to perform functional endoscopic sinus surgery (FESS). FESS is a minimally invasive surgical procedure where the sinus ostia are opened endoscopically to improve mucociliary transport and remove inflamed tissue (1, 5). While FESS is generally considered safe and effective, there can be potential complications associated with the procedure. The most dangerous complications include orbital injury and cerebrospinal fluid (CSF) leak. Another potential complication of FESS is infection or epistaxis (6).

Despite surgery, around 40% of the patients remain uncontrolled (7). For these patients, after further work up, several additional treatment options are available according to the EPOS guidelines: aspirin treatment after desensitization (ATAD), revision surgery, biologics or treatment with oral corticosteroids. In the past, most patients underwent a revision FESS. However, the revision procedure is more complex than the primary surgery due to the absence or alteration of anatomic landmarks, scar tissue, and bone thickening, increasing the risk of complications (8, 9). Moreover, revision surgery itself is also a potential risk factor for subsequent surgeries (10).

Since 2022, several biologics became reimbursed for the indication of CRSwNP and therefore available for our patients. Biologics are a specific kind of treatment with recombinant DNA-derived humanized monoclonal antibodies that selectively bind specific targets in the inflammatory cascade which contribute to the pathophysiology of CRSwNP (11, 12). Their efficacy and safety have been proven in various phase 3 trials (13–17). Reimbursement criteria differ across nations but mostly involve the following criteria matching the EUFOREA criteria for a biological: bilateral nasal polyps, a history of ESS, significant impact on quality of life, evidence of type 2 inflammation and need for rescue treatment despite appropriate maintenance treatment (11).

The impact of the availability of biologics and their reimbursement criteria on the number of revision ESS procedures for CRSwNP remains unexplored. This study aims to assess whether the introduction of biologics has influenced the frequency of revision FESS in CRSwNP patients.

## Materials and methods

### Study design

This academic retrospective observational study was conducted at the Department of Otorhinolaryngology, Head and Neck Surgery of the University Hospital Leuven, Belgium, and approved by the Research Ethics Committee on August 22, 2023. Patients undergoing bilateral ESS or DRAFIII operations for uncontrolled CRSwNP according to the EPOS criteria (1) and those who started biologic treatments for the indication of CRSwNP according to the EPOS criteria (1) between January 2019 and December 2023 were included. Collected patient characteristics included age, gender, number of ESS operations, allergies, aspirin intolerance, and smoking status.

### Data analysis

Data analysis was conducted using IBM SPSS Statistics (Version 27). Group differences were assessed with a two-tailed unpaired *t*-test or Mann–Whitney *U*-test, depending on normality. For comparisons across multiple groups, one-way ANOVA or the Kruskal–Wallis test with *post hoc* analysis was applied. Correlations between continuous variables were evaluated using Pearson's or Spearman's correlation, based on normality. Categorical variable differences were analyzed using the chi-squared test. Statistical significance was set at *p* < 0.05, with Bonferroni correction applied for multiple testing.

## Results

**Figure 1:**
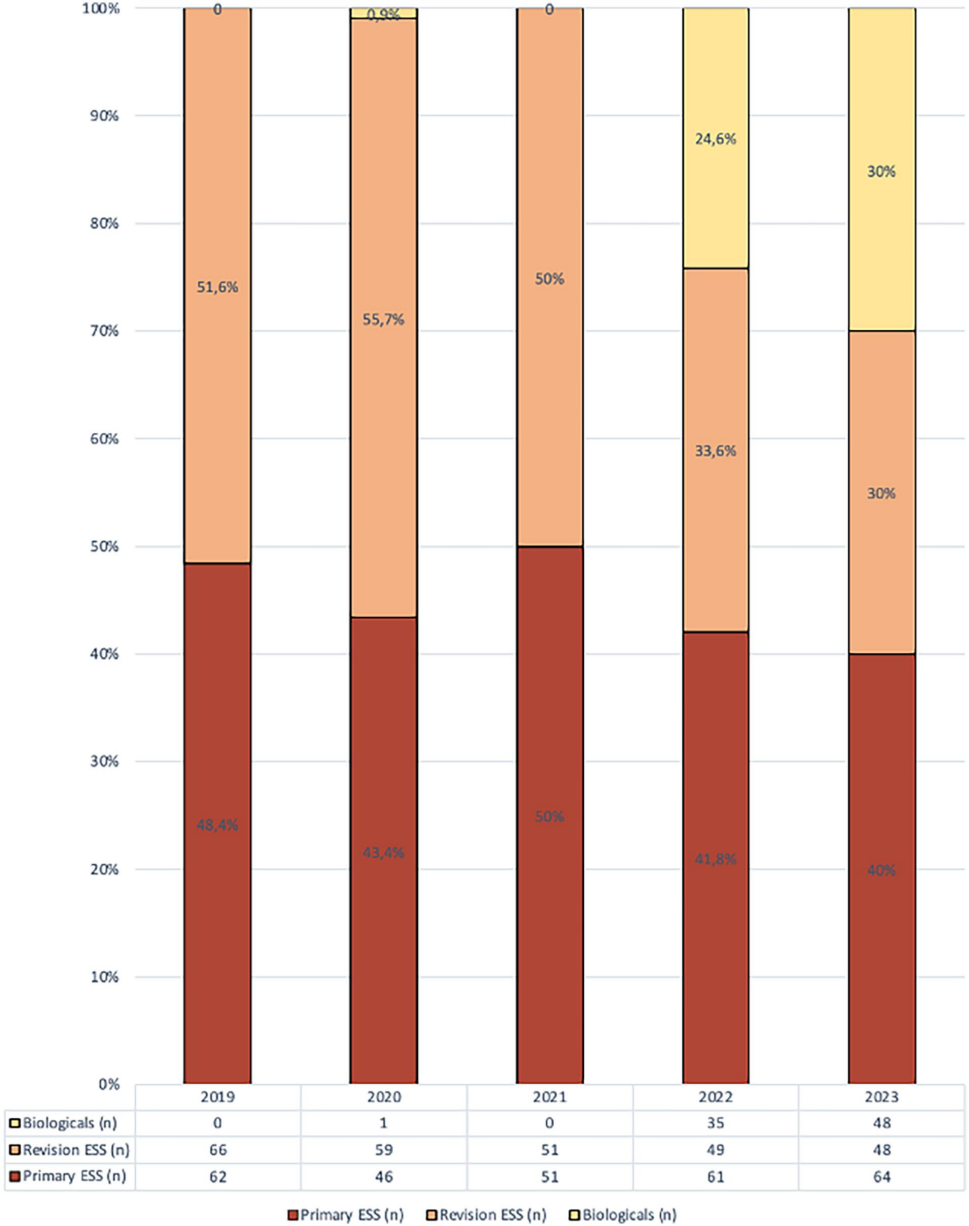
Percentages of the different treatments (biologicals, primary ESS, revision ESS) throughout the years (2019–2023).

A total of 641 patients with severe uncontrolled CRSwNP was offered primary/revision ESS or biologics between 2019 and 2023. Although there was a temporary decline in the total number of patients treated during the COVID-19 pandemic (2020–2021), a general upward trend was observed, with 128 patients treated in 2019 compared to 160 patients in 2023. The absolute number of patients undergoing revision ESS decreased over time, from 66 in 2019 and 59 in 2020 to 49 in 2022 and 48 in 2023. Similarly, the proportion of patients undergoing revision surgery also dropped from 52% in 2019% and 56% in 2020 to 34% in 2022% and 30% in 2023. In contrast, the absolute and relative number of primary ESS remained stable with 62 (48%) in 2019, 61 (42%) in 2022 and 46 (43%) in 2023 (Figure 1). Looking at differences in baseline characteristics. Patients undergoing a primary ESS were significantly younger (44.4 ± 17.5 years) compared to those undergoing revision ESS (48.1 ± 15.8 years) and those treated with biologics (52.4 ± 13.4 years). Patients in the biologics group had higher rates of comorbidities like allergies (61%), asthma (73%), and AERD (33.3%) compared to the primary and revision ESS groups, reflecting the high disease burden in these patients (Table 1).

**Table 1:** Patient characteristics.

Baseline characteristics	General (*n* = 641)	Primary FESS (*n* = 284)	Revision FESS (*n* = 273)	Biologicals (*n* = 84)
*Mean age *n* (± SD)	47 (±16.5)	44.4 (±17.5)	48.1 (±15.8)	52.1 (±13.4)
FESS *n* (%)				
- Primary	316 (49.3%)	284 (100%)	0 (%)	32 (48%)
- Revision	321 (50.1%)	0 (0%)	273 (100%)	48 (57.1%)
- None	4 (0.62%)	0 (0%)	0 (0%)	4 (4.7%)
*Allergy *n* (%)	288 (44.9%)	114 (40.1%)	123 (45.1%)	51 (60.7%)
*Asthma *n* (%)	246 (38.4%)	80 (28.1%)	105 (38.5%)	61 (62.6%)
*AERD *n* (%)	86 (13.4%)	9 (3.1%)	49 (17.9%)	28 (33.3%)
Smokers *n* (%)	86 (13.4%)	39 (13.7%)	40 (14.7%)	7 (8.3)
Ex-smokers	123 (19.2%)	54 (19.0%)	58 (21.2%)	11 (13.1%)

Significant differences between groups are indicated with a *.

## Discussion

To our knowledge, our study is the first to report the impact of the availability and reimbursement of biologics on the number of revision ESS in patients with CRSwNP (in a tertiary care center). Our study illustrates the regression in revision ESS numbers and percentages compared to primary ESS.

Throughout the 5 years, our study found a consistent decrease in the total number of FESS operations. Notably, a descending trend in the frequency of revision FESS exists, in contrast with a modest increase in primary FESS operations. The decline in primary FESS operations during the pandemic, particularly in 2020, can be attributed to various factors including reduced appointment numbers and delayed initiation of treatment pathways. Unlike primary procedures, the decrease in revision FESS was less. This can be attributed to pre-scheduled procedures and potentially higher urgency of cases. The slight increase in primary FESS could be either attributed to the impact of COVID-19 disruptions, prompting the rescheduling of postponed surgeries, or due to the need for at least one fess in order to be approved for reimbursement for biologicals in accordance with the EPOS/EUFOREA criteria (1, 11).

Our patient population and main findings were in line with current literature. The prevalence of asthma as a comorbidity in CRSwNP patients varies from 20% to 60% (1, 18), while the prevalence of allergies ranges around 36% (19). The prevalence of AERD in literature varies from 8% to 26%. (1, 18) In our study, the overall prevalence of AERD was 13.4%. When looking at the different cohorts, we see a significant difference between the three groups. The prevalence in the biological group (33.3%) was notably higher than in the revision FESS group, which was significantly higher than the primary FESS group, suggesting a greater necessity for revision surgery and biologicals among patients with AERD (*p* < 0.05, phi coefficient: 0.304). This hypothesis is in accordance with previous literature, showing that patients with comorbidities such as AERD or asthma, have more uncontrolled disease and, as a result, need more revision surgery (20). Another meta-analysis by Loftus et al. (10) showed that the rate of revision surgery in patients with comorbidities, such as asthma, is higher than the overall rate, indicating that patients with asthma have higher needs for revision surgery (10).

### Strengths and limitations

A key strength of this study is the large patient cohort, providing robust data on treatment trends over a five-year period. Furthermore, this is the first study to evaluate the impact of biologics on revision ESS numbers in a tertiary care setting. However, several limitations should be acknowledged. As a single-center study, our findings may not be fully generalizable to other healthcare settings as a selection bias may be present, as tertiary care centers often manage more severe cases of CRSwNP. Lastly, while our study suggests an association between biologics and declining revision ESS rates, causality cannot be definitively established without prospective studies.

### Future directions

To ascertain the persistence of the observed downward trend, it will be essential to conduct further investigations into FESS operations in subsequent years. A prospective study would allow for direct comparison between patients treated with biologics and those undergoing revision ESS, enabling a clearer evaluation of real-world treatment efficacy and patient outcomes. Future studies should also aim to include more detailed clinical variables, such as Lund-Mackay CT scores, SNOT-22 scores, and objective markers of type 2 inflammation, which could provide a more comprehensive understanding of disease severity and response to biologics vs. surgery.

## Conclusion

We can conclude that the reimbursement of the biologicals in Belgium reduced the number of revision FESS compared to primary FESS surgeries in patients with CRSwNP. Future longitudinal studies are warranted to further elucidate these trends.

## Data Availability

The raw data supporting the conclusions of this article will be made available by the authors, without undue reservation.
